# *Molinia caerulea* alters forest *Quercus petraea* seedling growth through reduced mycorrhization

**DOI:** 10.1093/aobpla/plac043

**Published:** 2022-09-29

**Authors:** Marine Fernandez, Philippe Malagoli, Lucie Vincenot, Antoine Vernay, Thierry Améglio, Philippe Balandier

**Affiliations:** Université Clermont Auvergne, INRAE, PIAF, F-63000 Clermont-Ferrand, France; Université Clermont Auvergne, INRAE, PIAF, F-63000 Clermont-Ferrand, France; Normandie Univ, UNIROUEN, Laboratoire ECODIV USC INRAE 1499, 76000 Rouen, France; Univ Lyon, Université Claude Bernard Lyon 1, CNRS, ENTPE, UMR 5023 LEHNA, F-69622 Villeurbanne, France; Université Clermont Auvergne, INRAE, PIAF, F-63000 Clermont-Ferrand, France; Université Clermont Auvergne, INRAE, PIAF, F-63000 Clermont-Ferrand, France

**Keywords:** Ectomycorrhization rate, fungal symbionts, interaction, lateral root, pot experiment

## Abstract

Oak regeneration is jeopardized by purple moor grass, a well-known competitive perennial grass in the temperate forests of Western Europe. Below-ground interactions regarding resource acquisition and interference have been demonstrated and have led to new questions about the negative impact of purple moor grass on ectomycorrhizal colonization. The objective was to examine the effects of moor grass on root system size and ectomycorrhization rate of oak seedlings as well as consequences on nitrogen (N) content in oak and soil. Oak seedlings and moor grass tufts were planted together or separately in pots under semi-controlled conditions (irrigated and natural light) and harvested 1 year after planting. Biomass, N content in shoot and root in oak and moor grass as well as number of lateral roots and ectomycorrhizal rate in oak were measured. Biomass in both oak shoot and root was reduced when planting with moor grass. Concurrently, oak lateral roots number and ectomycorrhization rate decreased, along with a reduction in N content in mixed-grown oak. An interference mechanism of moor grass is affecting oak seedlings performance through reduction in oak lateral roots number and its ectomycorrhization, observed in conjunction with a lower growth and N content in oak. By altering both oak roots and mycorrhizas, moor grass appears to be a species with a high allelopathic potential. More broadly, these results show the complexity of interspecific interactions that involve various ecological processes involving the soil microbial community and need to be explored *in situ*.

## Teaser

Interactions between plants can involve a wide variety of mechanisms. Oak regeneration failure in the presence of moor grass in European temperate forests demonstrated not only competition by exploitation but also interference and even facilitation, allowing the discovery of an antagonistic interaction. In this paper, we highlight a new target of the oak–moor grass interaction: oak ectomycorrhizae. We show that in the presence of moor grass, young oaks have a lower: (i) total biomass, (ii) number of lateral roots, (iii) ectomycorrhization rate and at the same time (iv) a lower nitrogen content than oaks placed without moor grass.

## Introduction

Competition from common perennial grasses has long been known to restrict tree seedling growth and survival during regeneration, whether natural or artificial (i.e. plantation) (Davis *et al.* 1998). Grass species can rapidly colonize large volumes of soil with their fast-growing fasciculate root systems (Freschet *et al.* 2017) and their efficient nutrient uptake (Coll *et al.* 2004; Picon-Cochard *et al.* 2006). Colonization by grasses depletes resources in forest soil, a process termed competition by resource exploitation (Bleasdale 1960). Competition for soil resources such as water and inorganic nitrogen (N) can stunt tree seedling growth and increase mortality rate (Gordon *et al.* 1989; Coll *et al.* 2003; Balandier *et al.* 2006), thus jeopardizing forest regeneration.

Competition detrimental to tree seedlings can also be mediated by interference. Interference results in the negative effect of a plant A on a plant B not by affecting the quantity of a resource, but by affecting the ability of plant B to access that non-limiting resource (Holdridge *et al.* 2016). For instance, tree seedlings root systems are found to be smaller in biomass and length due to the presence of graminoids (Harmer and Robertson 2003; Collet *et al.* 2006) and it has been demonstrated that, allelochemicals exuded by graminoids can cause neighbour trees growth inhibition (Fernandez *et al.* 2021). Such negative effects of graminoids on tree seedling root systems can affect water and nutrient uptake ability. This is the case in oak seedlings exhibiting a decreased concentration in both shoot and root resulting in a reduced N content when grown with annual and perennial graminoids like *Stipa pulchra*, *Avena barbata* or *Deschampsia cespitosa* (Welker *et al.* 1991; Cheng and Bledsoe 2004; Vernay *et al.* 2018a, b).

Below-ground competition by interference can also involve alteration of interactions of tree seedling roots with other organisms such as symbiotic fungi. Many studies have demonstrated that mycorrhizal symbiosis can favour tree seedling establishment, growth and productivity (Lu *et al.* 1998; Simard and Durall 2004; Javaid 2007; Richard *et al.* 2009; Van Der Heijden and Horton 2009; Walker and Mallik 2009; Bonfante and Genre 2010). The role of mycorrhizae in N uptake by tree seedlings is well-investigated at physiological and molecular levels (Chalot and Brun 1998; Govindarajulu *et al.* 2005; Chalot and Plassard 2011). In natural ecosystems (e.g. low anthropogenic impacts forests), mycorrhizae can provide up to 80 % of tree seedling N requirements (Van Der Heijden *et al.* 2008). In this context, many studies have demonstrated that grass can exert an inhibitory effect on mycorrhizal growth and association with tree seedlings, particularly for conifer species (Handley 1963; Robinson 1972; Inderjit and Mallik 1999; Inderjit and Callaway 2003; Mallik 2003; Mallik *et al.* 2016).

For instance, Nilsson *et al.* (1993) showed both root system growth, mycorrhizal fungus mycelium diameter (*Paxillus involutus*) and N uptake in *Pinus sylvestris* were affected by the Ericaceae *Empetrum hermaphroditum*. However, molecules involved in this interaction and their targets were not identified. Moreover, considering that evergreen conifer and deciduous trees have different functional traits involved in soil resource capture (root extension rate, root mass fraction, for instance) and use (N use efficiency [NUE], N content, for instance) (Tomlinson *et al.* 2019), it appears necessary to increase the knowledge of mycorrhizae-mediated interactions between grasses and deciduous tree seedlings.

The nature of the mycorrhization process may also differ according to plant ecological strategies: acquisitive species, that promote fast growth, resource acquisition and use, and biomass turnover (Reich 2014; Fernandez *et al.* 2022), are more likely to be colonized by arbuscular mycorrhizae than by ectomycorrhizae (Craig *et al.* 2018). Thus, the inhibitory effect of grass may not harm the two functional groups of trees, evergreen and deciduous, in the same way. Additionally, mycorrhizae can also negatively impact plant growth when the cost of symbiosis outweighs the benefits (Johnson *et al.* 1997; Jones and Smith 2004). Näsholm *et al.* (2013) demonstrated that positive feedback between decreased soil N availability and increased mycorrhizal N immobilization through increased below-ground carbon allocation may exacerbate N depletion in soils.

Such a negative effect of ectomycorrhizal association was also supported by Alberton *et al.* (2007), showing competition for N increases under elevated CO_2_. Other studies also explained that arbuscular mycorrhizal (AM) fungi are more likely to affect trees seedlings establishment than ectomycorrhizal fungi (EMF), especially in context of competition with grasses, due to common AM network existence between tree and grass (Alberton *et al.* 2007; Zangaro *et al.* 2018). The mycorrhization process therefore directly or indirectly, positively or negatively, affects plant dispersal and competition that shape plant populations and communities (Tedersoo *et al.* 2020).

Negative effects of a herbaceous plant on mycorrhization of a deciduous tree seedling were demonstrated by Timbal *et al.* (1990): They demonstrated neighbouring moor grass, *Molinia caerulea*, reduced red oak (*Quercus rubra*) growth 3- to 5-fold compared to sole-grown oak seedlings. They concomitantly found shifts in mycorrhizal biodiversity: biomass and/or proportion of efficient fungi (i.e. those involved in higher nutrient uptake ability) such as *Laccaria* sp. fell, while less efficient fungi such as *Cenococcum* sp. became more abundant. In some circumstances of low water or nutrient resources, the competition of *Molinia* on *Quercus*, especially *Quercus petraea*, can be problematic, compromising oak regeneration in Western Europe (Vernay *et al.* 2016). Our previous work has demonstrated strong competition for soil N in favour of moor grass, but nothing is known about interfering interactions targeting mycorrhizae and more broadly on the complexity of bidirectional interactions between plants.

For many years, work on plants interactions has mainly focused on competition (Grime 1974; Tilman 1990), generally described as unidirectional, i.e. negative effect of plant A on plant B without considering the effect of plant B on plant A. Callaway (1995) points out that facilitative interactions have been less studied because they would be ‘interesting but not convincing’ in plant communities. However, facilitation has gained interest since ecologists have shown that it can become the primary interaction when stress increases to some extent (Bertness and Callaway 1994; Michalet *et al.* 2006). According to the stress-gradient hypothesis, competition may also turn into facilitation when environmental conditions change (Pugnaire and Luque 2001; Maestre *et al.* 2009). Facilitation can also occur simultaneously with resource competition or allelopathy (Callaway 1995; Bronstein 2009; Schöb *et al.* 2014a, b) underlining the interest of considering interactions as a bidirectional process (Schöb *et al.* 2015). Recent studies showed that sessile oak (*Q. petraea*) improved grasses growth, evidencing an antagonistic interaction where reduced oak growth was concomitant with improved grass development (Vernay *et al.* 2018a; Fernandez *et al.* 2020). In this context we aimed at identifying the effect of moor grass on oak seedling mycorrhization and its effects on oak N uptake and biomass.

We investigated the interactions between *M. caerulea* (purple moor grass) and *Q. petraea* (sessile oak). *Molinia caerulea* is a grass with one of the strongest soil N uptake abilities (Heil and Bruggink 1987; Aerts and Berendse 1988; Persson and Nasholm 2001; Vernay *et al.* 2016). Sessile oak is a common species widespread in European temperate forests and is economically important (Eaton *et al.* 2016). We sought to determine whether competition between oak seedlings and moor grass involved not only exploitation of resources such as N, but also interference mechanisms affecting oak root system size and reducing ectomycorrhizal colonization of oak. We specifically hypothesized that moor grass (i) has a detrimental effect on the number of oak seedling lateral roots, and (ii) depresses ectomycorrhizal colonization of roots, resulting in (iii) a decrease in N uptake ability.

## Materials and Methods

### Plant material and experimental design

The experiment was conducted in pots under outdoor conditions in Clermont-Ferrand (Auvergne, France, 45°45ʹN 3°07ʹE, altitude 394 m a.s.l.) from April 2018 to May 2019 (mean temperature, *T*_mean_ = 12.9 °C, annual rainfall = 546 mm). An experiment in pots rather than under natural conditions was chosen to avoid confounding effects such as water availability. In April 2018, 12 one-year-old bare-root sessile oak seedlings and 12 moor grass tufts were planted in plastic pots, either separately or together. Oak seedlings were sourced from a local nursery (Pépinières Naudet, Leuglay, France). They weighed 32 ± 8.2 g (mean ± SE) (fresh), and measured 52 ± 5.8 cm in height, and 6.8 ± 0.9 mm in diameter. Moor grass (fresh weight 2.2 ± 0.9 g) was collected in a local forest at Paray-le-Frésil (Auvergne, France; 46°39ʹN 3°36ʹE); shoots and roots were cut at 6 cm each with 2.04 ± 0.97 g fresh weight per lot.

A total of six 10-L pots and twelve (grouped in pairs) 5-L pots were filled with soil (typical luvisol-redoxisol pseudogley, sandy loam) collected in the same forest as the moor grass tufts. Natural forest soil was used to preserve natural composition of EMF community. Two treatments based on root system separation or interaction were set up: (i) two separate 5-L pots containing either one oak seedling or one moor grass tuft were placed side-by-side, precluding all root interactions (‘sole-grown’ treatment) and (ii) one oak and one moor grass tuft were placed in a shared 10-L pot to allow full below-ground interactions through root and hyphae contacts (‘mixed-grown’ treatment). Each treatment was replicated six times with a random spatial pot arrangement. To avoid interaction with water availability, pots were irrigated to field capacity throughout the experiment. To ensure the correct water amount was delivered, soil water content was continuously measured with TDR probes with a target soil water content maintained between 15 and 30 % [see **Supporting Information—Figure S1** and **Appendix S1**]. No fertilizer was added to the pots during the experiment.

### Plant harvesting, lateral roots and ectomycorrhizal oak count

Plants were harvested in May 2019, after 13 months in pots to allow full mycorrhizal root colonization by fungi as well as interactions between plants. Shoots (stem + leaves together) and roots were collected for each plants. They were then dried at 60 °C for 48 h to measure shoot and root dry biomasses.

Immediately after root system harvest, approximately one-tenth of each oak total root system (fine and coarse roots) was stored in plastic bags at −20 °C for subsequent observation of lateral roots and occurrence of ectomycorrhizal root tips. For each treatment, 12 microscope slides were prepared, each slide harbouring ten 1-cm fragments of random primary fine roots (diameter < 2 mm) cut with a cutter was placed with water under a coverslip (20 fragments per root system of mixed-grown oak and 30 fragments per root system of sole-grown oak). The total of these 120 primary roots per treatment was observed with a binocular magnifier and Leica LAS Core® software with ×40 magnification. For each fine root fragment, the number of fine lateral roots and the number of ectomycorrhizal root tips were counted. Fungal species were not identified morphologically, but root tips were considered as ectomycorrhizal based on the distinctive presence of a hyphal sheath (the ectomycorrhiza mantle) surrounded with extramatrical hyphae **[see****Supporting Information—**Photo S1**]**. The rate of ectomycorrhization was then calculated as the number of ectomycorrhizal root tips/total number of observed root tips.

### Molecular identification of oak EMF

Ectomycorrhizal root tips were investigated with molecular barcoding when oak root systems were developed enough to apply the following procedure, that is, for six sole-grown oaks and only four mixed-grown oaks (insufficient root system with little or no branching with mycorrhizae). Under binocular magnifier and Leica LAS Core® software with ×40 magnification, 10 ectomycorrhizal root tips per oak root system were excised and stored at −20 °C until DNA extraction. For each excised ectomycorrhizal root tip, total DNA was extracted using DNeasy 96 Plant Pro Kit (Qiagen) following the supplier’s protocol. PCR amplification of locus *ITS* was performed using the primers ITS1-F (Gardes and Bruns 1993) and ITS4 (White *et al.* 1990) following PCR conditions described in Vincenot *et al.* (2012). Amplicons were purified using PCR DNA and Gel Band Purification Kit (GFX™), and Sanger-sequenced on ABI 3730 XL (Life Technologies/Thermofisher). Nucleotide sequences were visually inspected, then aligned and paired to retrieve contig sequences in MEGAX (Kumar *et al.* 2018). A total of 87 samples were sent for Sanger sequencing: 57 sequences of ectomycorrhizal roots of sole-grown oaks and 30 sequences of ectomycorrhizal roots of mixed-grown oaks. These sequences are available in GenBank database under accession numbers ON391357–ON391414. Eventually, taxonomic identity of fungi in ectomycorrhizal root tips was retrieved by submitting contig sequences to BLAST search in fungi molecular database UNITE (Nilsson *et al.* 2019).

### Plant N content

After drying at 60 °C for at least 48 h, shoots and roots were weighed and ground to a fine powder. Total N content (% of dry weight) was then determined with an elemental analyser (vario ISOTOPE cube, Elementar, Hanau, Germany) in line with a gas isotope ratio mass spectrometer (IsoPrime 100, Isoprime Ltd, Cheadle, UK) at the analytic platform SilvaTech.

### Statistics

Statistical analysis was performed using R software (Version 3.4.1). Data were means of *n* = 6 plants for dry weight in each treatment, and *n* = 120 pseudo-replicates (*n* = 20 fragments per root system of the total six mixed-grown oak and *n* = 30 fragments per root system of the total four sole-grown oak) for lateral root tips and ectomycorrhizal root tip counts. All variables were tested for normality and homoscedasticity, using the Shapiro−Wilk and Levene tests. Data analysed using ANOVA. Data of lateral root tips and ectomycorrhizal root tip counts were analysed using linear models (lmer function), where treatment was the predictor variable and oak identity was a random effect.

## Results

**Figure 1.**
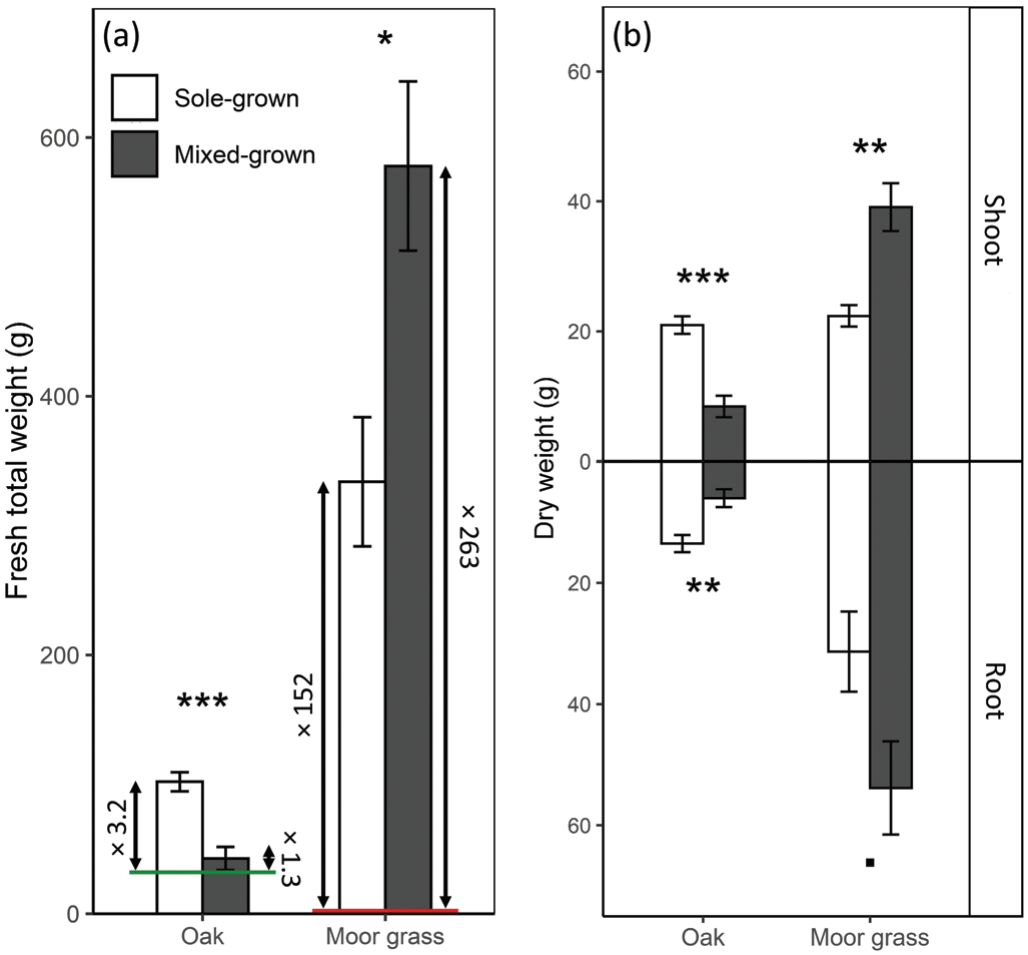
(A) Fresh total weight of oak and moor grass when sole-grown (white) or mixed-grown (grey). The two horizontal lines indicate the total fresh weight at the beginning of experiment for oak and moor grass, respectively. The numbers indicate the increase in biomass between the beginning of the experiment and the harvest. (B) Dry weight of oak and moor grass shoots and roots. Values are reported as means ± SE. *, **, *** correspond to *P* < 0.05, 0.01 and 0.001, respectively, for Student’s *t*-test for each organ, *n* = 6.

Mean total oak dry weight increased by 3.2-fold between the planting (April 2018) and the harvest (May 2019) for sole-grown oaks, compared with only 1.3-fold for mixed-grown oaks with moor grass (Fig. 1A; **see****Supporting Information—**Table S1). Both shoot (stem + leaves) and root biomass showed 2.5- and 2.2-fold decreases, respectively, when mixed-grown with moor grass (*P* < 0.001 and *P* = 0.011, respectively; Fig. 1B; **see****Supporting Information—Table S2**). Whether sole-grown or grown in mix, moor grass grew much faster than oak: between April 2018 and May 2019, mean moor grass dry weight increased over 150 times when sole-grown, compared with over 260 times when mixed-grown with oak (Fig. 1A; **see****Supporting Information—Table S1**). Conversely to oak, moor grass exhibited a higher (1.7-fold increase) mean dry weight when mixed-grown than when sole-grown for both shoot and root (*P* = 0.005 and *P* = 0.052, respectively; Fig. 1B; **see****Supporting Information—Table S2**).

**Figure 2.**
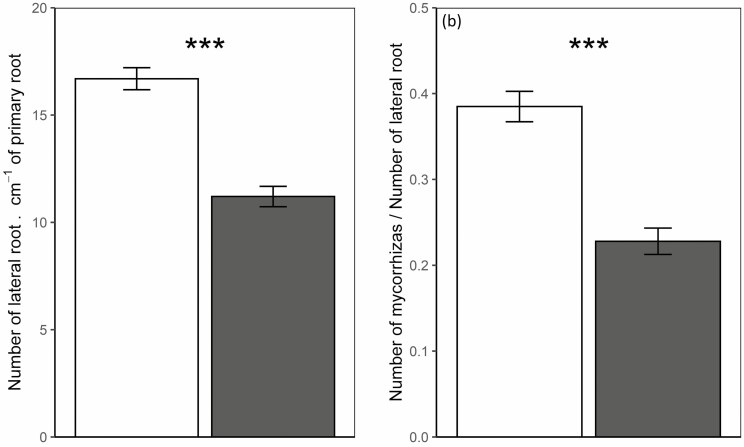
Number of lateral roots (A) and ectomycorrhization rate, i.e. ratio of number of mycorrhizal root tips to number of lateral roots (B) when sole-grown (white) or mixed-grown (grey). Values are reported as means ± SE. *** corresponds to *P* < 0.001 for Student’s *t*-test for each organ, *n* = 120.

Sole-grown oak roots displayed a mean number of 16.7 fine lateral roots per cm (Fig. 2A; **see****Supporting Information—Table S3**), with an average of 6.6 ectomycorrhizal root tips per cm (Fig. 2B; **see****Supporting Information—Table S4**), thus about 40 % of root tips having formed ectomycorrhizal association. When mixed-grown with moor grass, both the numbers of oak fine lateral roots (11.2·cm^−1^) and ectomycorrhizal root tips (2.8·cm^−1^) were markedly reduced (*P* < 0.001 for both root tips and ectomycorrhizal root tips), leading to a 2.4-fold decrease of oak ectomycorrhization rate.

**Figure 3.**
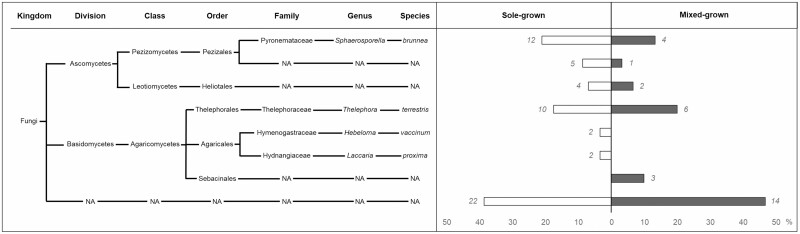
Proportion in percentage of oak root mycorrhizas in sole-grown (white) and mixed-grown (grey) for each taxon. Italic number next bar indicated total number of mycorrhizas sequences. The results of BLAST analyses assigning taxa to each sequence are shown on the left. NA means that the BLAST result did not give more details about the taxon. The last line represents the sequences that did not give any BLAST result.

Molecular typing of ectomycorrhizal root tips allowed the identification of 35 sequences among the 57 sequences in sole-grown oaks and 16 sequences among the 30 sequences in mixed-grown oaks, the rest remaining as ‘unidentified taxa’ after BLAST due to poor-quality sequence. Six different taxa were identified in sole-grown versus five in mixed-grown treatment (Fig. 3). In sole-grown, Ascomycota was more abundant (36.8 %) than Basidiomycota (24.6 %) on the contrary to mixed-grown (23.3 % and 30 % of Ascomycota and Basidiomycota, respectively). *Sphaerosporella brunnea* (Ascomycota) and *Thelophora terrestris* (Basidiomycota) were present in both treatments, with a higher proportion of *S. brunnea* (21.1 %) than *T. terrestris* (17.5 %) in sole-grown treatment. Conversely, in mixed-grown the proportion of *S. brunnea* (13.3 %) was lower than of *T. terrestris* (20 %). Mycorrhizal fungi of the orders Heliotales and Pezizales were also present in both treatments (7.0 % and 6.7 % in sole-grown and mixed-grown, respectively, for Heliotales; 8.8 % and 3.3 % in sole-grown and mixed-grown, respectively, for Pezizales), yet their families could not be identified. *Laccaria proxima* and *Hebeloma vaccinum* were also detected in sole-grown treatment only, in lower proportions (3.5 % for each of these). In mixed-grown, 10 % of Sebacinales were identified, but none in sole-grown. Unidentified sequences represent 38.6 % in sole-grown and 46.7 % in mixed-grown.

**Figure 4.**
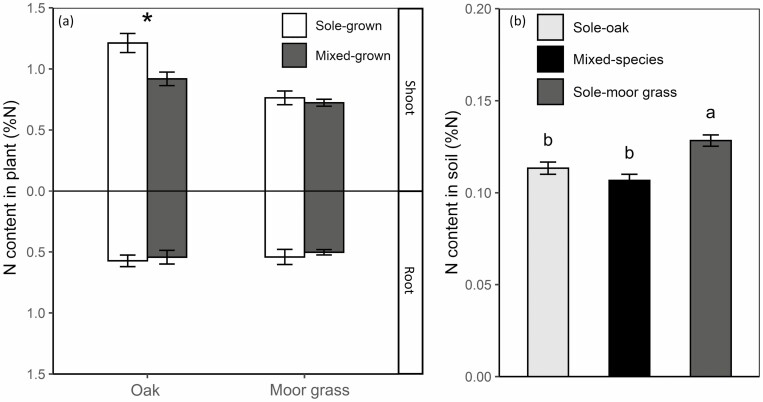
Nitrogen content in plant (A) shoots and roots when sole-grown (white) or mixed-grown (grey) and in soil (B) in sole-oak pot (light grey), sole-moor grass pot and (dark grey) in mixed-species pot (black). Values are reported as means ± SE. *correspond to *P* < 0.05, for Student’s *t*-test for each organ, *n* = 6.

Along with the decrease in oak biomass when mixed-grown, there was a decrease in oak shoots N content when mixed-grown moor grass (*P =* 0.014; from 1.2 % of dry shoot weight in sole-grown to 0.9 % in mixed-grown oak; Fig. 4A; **see****Supporting Information—Table S5**) but N content in oak root was not changed in mixed-grown (*P* = 0.69; 0.57 % and 0.54 % of dry root weight when sole-grown vs. mixed-grown, respectively). Nitrogen content in soil was statistically similar between pots containing sole-oak and pots containing mixed-grown (*P* = 0.340), but significantly higher in pots containing sole-moor grass (*P* = 0.014 and *P* < 0.001 for sole-oak vs. sole-moor grass and mixed-species vs. sole-moor grass, respectively) (Fig. 4B; **see****Supporting Information—Tables S6** and **S7**).

## Discussion

### Moor grass competition impairs oak root ability to forage for N

Presence of moor grass in the same pot as oak seedlings resulted in reduced growth of oak compared to sole-grown seedlings, as shown by a lower biomass production. Also, oak root branching (number of lateral fine roots/fine root length) lowered when mixed-grown with moor grass. These results are consistent with those of other studies reporting the negative effect of grass species on the number of lateral roots, leading to decreased tree seedling growth (Schroth 1999; Harmer and Robertson 2003; Schaller *et al.* 2003; Collet and Chenost 2006). Root foraging ability associated with root system architecture, and particularly branching, plays a critical role in nutrient uptake, particularly of N and phosphorus (Bar-Tal *et al.* 1997; Postma *et al.* 2014; Shahzad and Amtmann 2017; Duque and Villordon 2019). Profuse branching of root systems increases both soil exchange surfaces and the volume of soil that can be explored (Fitter *et al.* 1991). Some studies have demonstrated that a localized supply of nutrients increases lateral root density and elongation only where the nutrient levels are high (Hackett 1972; Drew and Saker 1975; Granato and Raper 1989; Hodge 2004), yet a homogenous nutrient deficiency can stimulate lateral root formation to increase soil exploitation (Shahzad and Amtmann 2017). Conversely, others have observed an inhibitory effect of high nutrient concentration on root growth (Zhang and Forde 1998; Zhang *et al.* 1999; Linkohr *et al.* 2002; Celis-Arámburo *et al.* 2011; Gruber *et al.* 2013).

In our experiment, N availability in soil probably does not explain lateral root inhibition, as N content values were similar in sole- and mixed-grown treatments (N content was 0.13 ± 0.003 % and 0.11 ± 0.003 % in sole-grown and mixed-grown, respectively, without statistically significant difference). However, numerous other factors have been identified as inhibitors of lateral root formation and growth, including phytohormones (Fukaki and Tasaka 2009; Shkolnik-Inbar and Bar-Zvi 2010; Lewis *et al.* 2011). In the specific context of a mixed-grown treatment, shoot and root extracts of some species, generally grass and crops, can have an allelopathic effect on lateral root formation and length in neighbours (Horsley 1977; Pardales *et al.* 1992; Chon *et al.* 2002; Amoo *et al.* 2008; Hossain *et al.* 2016). Ahmed *et al.* (2007) reported such an inhibitory effect was much more pronounced in root and lateral root development than for shoot or seed germination. This suggests inhibition of oak root system development by moor grass could play a key role in the interaction between the two species.

### Moor grass depletes oak root ectomycorrhization

Moor grass also reduced ectomycorrhizal association rate with oak roots, as the number of ectomycorrhizal root tips was 1.9 times lower when mixed-grown with moor grass. To our knowledge, such a reduction of association rate with EMF by moor grass has not been reported yet.

Regarding mechanisms that can inhibit mycorrhizal colonization, most studies focused on vesicular AM symbiosis (Abbott and Gazey 1994; Koide and Schreiner 2003; Garg and Chandel 2010). As for lateral root initiation and development, edaphic parameters can inhibit root association with EMF, such as phosphorus enrichment, low soil temperature or phytohormones (Graham *et al.* 1982; el Ghachtouli *et al.* 1996; Javaid 2008; Kobae *et al.* 2016). Allelochemical exudation by roots may also be involved in the inhibition of both endomycorrhizae and ectomycorrhizae, but only a few studies have addressed that mechanism, mostly testing for allelopathy using leaf extracts at various concentrations (Olsen *et al.* 1971; Nilsson 1994; Javaid 2007, 2008). In this line, Nilsson *et al.* (1993) suggested that a decrease in ectomycorrhizal symbiosis of *P. sylvestris* seedlings was probably due to a direct negative effect of *E. hermaphroditum* on hyphal diameter. Pellissier (1994) identified phenolic allelochemicals in forest floor humus involved in conifers (*Picea abies* and *Picea mariana*) regeneration failure, possibly because these compounds can directly reduce root growth and ectomycorrhizal formation (Mallik 1987, 1998, 2003; Zhu and Mallik 1994; Inderjit and Mallik 1996).

An allelopathic activity of moor grass has been suggested in a few studies (Becker and Lévy 1982; Timbal *et al.* 1990), but no allelopathic compound has been clearly identified so far (Fernandez *et al.* 2021). For instance, Timbal *et al.* (1990) showed no impact of moor grass on fungal taxonomic richness but a change of species community composition of EMF in *Q. rubra* seedlings roots, suggesting the necessity of studying composition of *Molinia* roots exudates. Moor grass roots and shoots might contain phenolic compounds that can be released into the soil through litter decomposition, leaching and exudation, with possible inhibitory effects on oak lateral root initiation, mycorrhizal symbiosis establishment or functioning, or directly on fungal hyphae development. Allelopathic compounds of moor grass may also act as an environmental filter on the structure of ectomycorrhizal communities associated to oak roots (Becker and Lévy 1982; Timbal *et al.* 1990; Fernandez *et al.* 2021).

### Number of oak EMF sequences decreases when mixed-grown with moor grass

Analyses of molecular identification of EMF confirmed oaks mixed-grown with moor grass harboured lower numbers of sequenced mycorrhizal taxa than sole-grown oaks. However, caution should be exercised in interpreting the effect of moor grass on oak mycorrhizal biodiversity because 38.6 % and 46.7 % of sequences could not be identified in sole-grown and mixed-grown, respectively. Unidentified fungal sequences represent 17.3 % in sole-grown and 40.7 % in mixed-grown. Such a difference can be explained by a more necrotic root system that was observed in mixed-grown during harvest, and thus this could lead to lower quality ectomycorrhizal samples. Because oaks mixed-grown with moor grass had a lower root system biomass and a lower mycorrhization rate than sole-grown oaks, it was then more difficult to harvest mycorrhizae and extract DNA.

Moreover, Timbal *et al.* (1990) demonstrated a change in EMF community composition but not in abundance. Vořiškova *et al.*’s (2014) study on *Q. petraea* showed that Basidiomycota was the dominant division, in line with our results only in mixed-grown oak. Also, in temperate forest, Basidiomycota are commonly more represented than Ascomycota (Venturella *et al.* 2011; Weber *et al.* 2013), especially under *Quercus* spp. (Morris *et al.* 2008; Smith *et al.* 2009; Toju *et al.* 2013a, b). Nevertheless, He *et al.* (2016) measured a higher proportion of Ascomycota (64.6 %) than Basidiomycota (26.6 %) on *Quercus mongolica* root system, in accordance with our study in sole-grown treatment. Weber *et al.* (2013) demonstrated that the proportion of Ascomycota in bulk soil increases with soil depth with a corresponding decrease in Basidiomycota. Here, we harvested mycorrhizae from the entire root system, not just from the surface, which possibly partly explains a higher proportion of Ascomycota than Basidiomycota in sole-grown treatment in comparison with other studies where roots were harvested in the surface soil only (0–15 cm). Other drivers may explain an unexpected proportion of Ascomycota in ectomycorrhizal fungal community, such as seedlings or trees origin and age, soil properties and environmental conditions (Morris *et al.* 2008; Wu *et al.* 2013; Lamit *et al.* 2016). In the studies cited above, all samples were taken from mature trees located in forests, whereas we worked with oak nursery seedlings in a pot experiment.

Sebacinales were only found in mixed-grown oak roots, but possibly this taxon was also present among the unidentified sequences in sole-grown oaks. Sebacinales are commonly found on the roots of many dicot and monocot plant species (Weiß *et al.* 2016), including oaks (Richard *et al.* 2011; Oberwinkler *et al.* 2013), and participate to common mycorrhizal networks that can allow nutrients exchanges amongst host plants (Selosse *et al.* 2017). Potentially shared Sebacinales raise the hypothesis of C transfer between oak and moor grass through shared mycorrhizal symbiotic mycelia, which would need further isotopic investigation to be verified. *Hebeloma vaccinum* and *L. proxima* were only found in sole-grown oak and also commonly form ectomycorrhizae with *Quercus* sp. (Leski *et al.* 2010; Otsing and Tedersoo 2015). We demonstrated the strong negative effect of moor grass on relative numbers of retrieved EMF sequences, underlying the importance of mycorrhizae identification to describe interactions between plants. Our results suggest possible allelochemical compounds of moor grass may filter oak EMF community composition and impair ectomycorrhizal establishment in oak root system, limiting oak growth (Fernandez *et al.* 2021). The specific intensity of moor grass negative effects on EMF taxa can also be discussed. Relative numbers in sequenced EMF community were lower on mixed-grown oaks than on sole-grown oaks for EMF various taxa (Pezizales 2.6 times lower; *S. brunnea*, 1.6 times lower; Helotiales 1.1 time), suggesting a fungal-specific variation of intensity of moor grass negative effect. Such shifts in the EMF taxa relative numbers of EMF community were consistent with other studies demonstrating disproportional impact of allelochemicals on mycorrhizal fungi (Roche *et al.* 2021).

### Oaks N allocation: the conservative strategy

Along with a decrease in shoot dry weight, the significantly lower N content in oak shoot for mixed-grown strongly suggests a reduced ability to take soil N up, associated with the decrease in lateral roots and ectomycorrhization as discussed above. In sole-grown treatment, mean N content in oak shoot was 1.21 ± 0.08 %, in line with previous findings (Espelta *et al.* 2005), but when oak was mixed-grown with moor grass, mean N content was only 0.92 ± 0.05 %.

Nitrogen content in oak roots was similar in the two treatments (Fig. 4B), although dry weight was lower when mixed-grown, indicating N allocation towards root in oaks with moor grass was larger than in sole-grown oaks. These results are consistent with previous findings highlighting a modification of N allocation in forest tree seedlings in the presence of grasses (Welker *et al.* 1991; Coll *et al.* 2004). Nitrogen allocation to roots did not correlate with investment in lateral root development, prospection and resource capture, so it strongly suggests a strategy for N storage (i.e. conservative). According to Grime (1974), moor grass, through its strong competitive strategy, induces stress in oak seedling resulting in intensification of N conservative strategy and a lower ability to compete for N soil. Vernay *et al.* (2018b) also described modification of N allocation in oak seedling when mixed-grown with *D. cespitosa*, whereas fine root dry weight was constant. This confirms a different conservative strategy of N economy due to the perturbation caused by neighbouring grass. One possibility is that limiting root growth and biomass will maintain sufficient N for plant survival.

Nitrogen content in soil was unexpectedly higher in pot containing sole-grown moor grass compared to pot containing sole-grown oaks or pot with the mixed-grown association. This result was not consistent with other study demonstrating that grass significantly decrease nitrate amount—but not ammonium—n soil compared to oak seedling (Vernay *et al.* 2018a). Although N content was higher in soil of sole-grown moor grass compared to mixed-grown, N content was statistically similar in both moor grass shoot and root. Difference of N content between shoot and root did not corroborate difference of biomass which was much higher in mixed-grown. It questions the effect of the presence of a neighbour on the plant NUE (Congreves *et al.* 2021): do oak seedlings favour moor grass NUE? Beyond N allocation and use, competition for soil N between oak and moor grass refers to theories of interspecific competition regarding resource use and availability. According to Tilman (1990) R* rule, R* (i.e. the lowest minimum resource threshold tolerated by a given species) of moor grass is supposed to be lower than oak R* suggesting that its competitive potential is greater as soil N availability decreases. However, contrary to Tilman’s theory as well as game theory (Mcnickle and Dybzinski 2013), competitive potential for soil resource of moor grass was not expressed by higher root biomass, but by higher shoot biomass. These suggestions also question the tragedy of the commons indicating that inter-plant competition for shared space should lead to increased root biomass and forage but decreased yield (Gersani *et al.* 2001). On the contrary, our study demonstrates that in a context of interspecific competition, a Poaceae can show a small increase in its root biomass but a clear increase in its aerial biomass. The challenge in fitting our results with existing ecological theories probably stems from the fact that the oak–molinia interaction is antagonistic, which is still poorly documented.

### Oak increased moor grass biomass: the antagonistic interaction

Higher biomass production of moor grass when mixed-grown with oak observed here is consistent with previous results demonstrating facilitative effects of oak seedlings on grasses (Vernay *et al.* 2018a) due to rapid and significant N transfer from oak to grass (Fernandez *et al.* 2020). In the complex framework of plant interactions, our results raise the following questions: (i) Is the competitive potential of the moor grass enhanced in the presence of oak seedlings because of intensification for N competition according to Tilman’s R* theory? (ii) Does the competitive potential of the moor grass increase the stress in oak seedlings resulting in a decrease of N competition according to Grime’s theory? And then, (iii) can we really talk about antagonistic interaction or modification of competitive potential of moor grass in the presence of oak seedlings? Our results perfectly corroborate Callaway’s (1995) statement: ‘the overall effect of one species on another may be the product of multiple, complex interactions’. Interactions between these two species constitute a double jeopardy for oak, which is stunted by moor grass and has been also shown here to facilitate moor grass biomass.

### Consequences of the interference interactions in natural ecosystems

Our experiment needs to be replicated *in situ*, as growth in pots can modify root architecture compared with naturally regenerated oak seedlings (Tsakaldimi *et al.* 2009), and impacts on ectomycorrhizal rate could be altered due to reduced space in the pot. The role of soil bacteria modulating N provision in the rhizosphere could be studied to gain a better understanding of the mechanisms by which lateral root formation and ectomycorrhization are inhibited. Then, in order to better characterize the interactions and question the ecological theories on plant competition, it would be interesting to measure the amount of nutrients (N, C, P, K) in the soil throughout the experiment. In general, analyses of plant, soil nutrients and mycorrhizal content would help clarify the processes and bio-actors targeted by the moor grass.

Furthermore, a common AM network can enhance inter- or intraspecific transfer of allelochemicals directly to the root system of the target species in pot experiment (Barto *et al.* 2011). Several oak species appear as dual host trees that can associate both with ectomycorrhizal and AM fungi (Dickie *et al.* 2004; He *et al.* 2006; Toju *et al.* 2013a, b). The latter being typically connected to herbaceous host plants, potential allelopathic effects of moor grass may be relayed by mycorrhizal symbionts shared with oak seedlings. This hypothesis could be further investigated by searching for shared AM fungal species and/or genotypes in moor grass and oak root systems. Also, the impacts of oak mycorrhizal communities’ alteration by moor grass on N uptake decrease would deserve further investigation. Eventually, our findings suggesting a strong allelopathic effect of moor grass on oak seedlings call for the identification of phenolic compounds present in moor grass root exudates.

In oak–moor grass stands, it has been clearly demonstrated that thinning induces a large influx of light into the understory favouring the development of the moor grass and hinders the regeneration of the oak (Vernay *et al.* 2016). However, the understory vegetation often appears to be essential to the forest renewal process, by promoting nutrient turnover and thus produce more fertile soils than bare soils (Watt 1919; Bobiec *et al.* 2018). Forest management must therefore consider the balance between thinning intensity and the consequences on the interactions between understory species and seedlings.

## Supplementary Material

plac043_suppl_Supplementary_Appendix

plac043_suppl_Supplementary_Figure_S1

plac043_suppl_Supplementary_Photo_S1

plac043_suppl_Supplementary_Tables

## Data Availability

The data sets generated during and/or analysed for the present study are available from the corresponding author on reasonable request. Sequences produced for molecular identification of fungal mycorrhizal symbionts are deposited in GenBank under accession numbers ON391357–ON391414.

## Notes

**Populations & Communities. Chief Editor:** Jean Burns
