# Supplementary material for: Molinia caerulea alters forest Quercus petraea seedling growth through reduced mycorrhization
Source: AoB Plants. 2022 Sep 29;15(2):plac043. doi: 10.1093/aobpla/plac043 (PMC9893876; doi:10.1093/aobpla/plac043)
Supplement: plac043_suppl_Supplementary_Appendix [file plac043_suppl_supplementary_appendix.docx]

**Appendix A1. Evolution of soil water content in the pots**

To avoid interaction with confounding factors in the experiment such as water availability, pots were irrigated to field capacity throughout the experiment. To ensure the correct water amount was delivered, volumetric soil water content was continuously measured with TDR probes in a previous experiment (2017 – 2018), allowing to test the optimal water supply for this experiment (Figure S1). The hydric characteristics of the soil were determined by establishing a curve between volumetric soil water content and matrix potential and gave 22.5% for field capacity and 9.4% for permanent wilting point. Figure S1 shows that most of time pots were at field capacity and experienced only slight droughty periods (values below the red line, 40% of soil water holding capacity, Vicca et al. 2012), too short to influence results reported in that study. In particular at the end of the experiment, we stopped irrigation 2 weeks before plant harvest to facilitate root extraction from the soil. Exactly the same water supply was added in 2018–2019 experiment.
