# Supplementary material for: Molinia caerulea alters forest Quercus petraea seedling growth through reduced mycorrhization
Source: AoB Plants. 2022 Sep 29;15(2):plac043. doi: 10.1093/aobpla/plac043 (PMC9893876; doi:10.1093/aobpla/plac043)
Supplement: plac043_suppl_Supplementary_Tables [file plac043_suppl_supplementary_tables.docx]

**Supplementary tables of**

**“*****Molinia caerulea* alters forest *Quercus petraea* seedling growth through reduced mycorrhization”**

**Table S1. (Figure 1.a).** Means ± SE of oak and moor grass fresh total weight in sole-grown (SG) and mixed-grown (MG) treatment. Test statistic (*F*-value), statistical significance (*p*-value), and degrees of freedom (DF) assessing the effect of **treatment** on oak and moor grass (*n* = 6).

|  | **Oak SG** | **Oak MG** | **Moor grass SG** | **Moor grass MG** |
| --- | --- | --- | --- | --- |
| Mean | 101.96 ± 7.28 | 42.71 ± 8.90 | 333.92 ± 49.94 | 578.00 ± 65.41 |
| P-value | 0.0004 | | 0.014 | |
| F-value | 26.55 | | 8.797 | |
| DF | 1 | | 1 | |

**Table S2. (Figure 1.b).** Means ± SE of oak and moor grass dry weight in sole-grown (SG) and mixed-grown (MG) treatment. Test statistic (*F*-value), statistical significance (*p*-value), and degrees of freedom (DF) assessing the effect of **treatment** on oak and moor grass (*n* = 6).

|  |  | **Oak SG** | **Oak MG** | **Moor grass SG** | **Moor grass MG** |
| --- | --- | --- | --- | --- | --- |
| **Shoot** | Mean | 20.94 ± 1.36 | 8.41 ± 1.67 | 22.35 ± 1.64 | 39.10 ± 3.69 |
|  | P-value | 0.0002 | | 0.002 | |
|  | F-value | 33.96 | | 17.17 | |
|  | DF | 1 | | 1 | |
| **Root** | Mean | 13.50 ± 1.43 | 6.02 ± 1.49 | 31.34 ± 6.61 | 53.83 ± 7.70 |
|  | P-value | 0.005 | | 0.05 | |
|  | F-value | 13.1 | | 4.91 | |
|  | DF | 1 | | 1 | |

**Table S3. (Figure 2.a).** Means ± SE of oak and moor grass number of lateral root per cm of primary root in sole-grown (SG) and mixed-grown (MG) treatment. Test statistic (*F*-value), statistical significance (*p*-value), and degrees of freedom (DF) assessing the effect of **treatment** and oak idendityon oak (*n* = 120).

|  |  | **Oak SG** | **Oak MG** |
| --- | --- | --- | --- |
|  | Mean | 16.69 ± 0.51 | 11.21 ± 0.47 |
|  | P-value | < 0.001 | |
| Treatment | F-value | 65.30 | |
|  | DF | 1 | |
| Oak identity | P-value | 0.001 | |
|  | F-value | 3.10 | |
|  | DF | 9 | |

**Table S4. (Figure 2.b).** Means ± SE of oak and moor grass number of mycorrhizaes/number of lateral root in sole-grown (SG) and mixed-grown (MG) treatment. Test statistic (*F*-value), statistical significance (*p*-value), and degrees of freedom (DF) assessing the effect of **treatment** and oak identity on oak (*n* = 120).

|  |  | **Oak SG** | **Oak MG** |
| --- | --- | --- | --- |
|  | Mean | 0.38 ± 0.02 | 0.23 ± 0.02 |
|  | P-value | < 0.001 | |
| Treatment | F-value | 52.28 | |
|  | DF | 1 | |
| Oak identity | P-value | < 0.001 | |
|  | F-value | 6.11 | |
|  | DF | 9 | |

**Table S5. (Figure 4.a).** Means ± SE of oak and moor grass N content in sole-grown (SG) and mixed-grown (MG) treatment. Test statistic (*F*-value), statistical significance (*p*-value), and degrees of freedom (DF) assessing the effect of **treatment** on oak and moor grass (*n* = 6).

|  |  | **Oak SG** | **Oak MG** | **Moor grass SG** | **Moor grass MG** |
| --- | --- | --- | --- | --- | --- |
| **Shoot** | Mean | 1.21 ± 0.08 | 0.92 ± 0.06 | 0.76 ± 0.06 | 0.72 ± 0.03 |
|  | P-value | 0.01 | | 0.54 | |
|  | F-value | 9.38 | | 0.40 | |
|  | DF | 1 | | 1 | |
| **Root** | Mean | 0.57 ± 0.05 | 0.54 ± 0.06 | 0.54 ± 0.06 | 0.50 ± 0.02 |
|  | P-value | 0.69 | | 0.57 | |
|  | F-value | 0.17 | | 0.34 | |
|  | DF | 1 | | 1 | |

**Table S6. (Figure 4.b).** Means ± SE of sole-oak, mixed species and sole-moor grass N content in soil (*n = 6*).

|  | **Sole-oak** | **Mixed species** | **Sole-moor grass** |
| --- | --- | --- | --- |
| **Mean** | 0.11 ± 0.003 | 0.11 ± 0.003 | 0.13 ± 0.003 |

**Table S7. (Figure 4.b).** P-value of sole-oak, mixed species and sole-moor grass N content in soil assessing the effect of **treatment** on oak and moor grass (*n* = 6).

|  | **Sole-oak / Mixed-species** | **Sole-oak / Sole-moor grass** | **Sole-moor grass / Mixed species** |
| --- | --- | --- | --- |
| **P-value** | 0.34 | 0.01 | 0.0008 |
